# Wild Birds and Urban Ecology of Ticks and Tick-borne Pathogens, Chicago, Illinois, USA, 2005–2010

**DOI:** 10.3201/eid1810.120511

**Published:** 2012-10

**Authors:** Sarah A. Hamer, Tony L. Goldberg, Uriel D. Kitron, Jeffrey D. Brawn, Tavis K. Anderson, Scott R. Loss, Edward D. Walker, Gabriel L. Hamer

**Affiliations:** Michigan State University, East Lansing, Michigan, USA (S.A. Hamer, E.D. Walker, G.L. Hamer);; Texas A&M University, College Station, Texas, USA (S.A. Hamer, G.L. Hamer);; University of Wisconsin, Madison, Wisconsin, USA (T.L. Goldberg, T.K. Anderson);; Emory University, Atlanta, Georgia, USA (U.D. Kitron); University of Illinois, Urbana, Illinois, USA (J.D. Brawn, S.R. Loss);; and Smithsonian Migratory Bird Center, Washington, DC, USA (S.R. Loss)

**Keywords:** ticks, birds, tick-borne diseases, tick-borne pathogens, Borrelia burgdorferi, bacteria, zoonoses, urban ecology, urbanization, Chicago, vector-borne infections, United States

## Abstract

Bird-facilitated introduction of ticks and associated pathogens is postulated to promote invasion of tick-borne zoonotic diseases into urban areas. Results of a longitudinal study conducted in suburban Chicago, Illinois, USA, during 2005–2010 show that 1.6% of 6,180 wild birds captured in mist nets harbored ticks. Tick species in order of abundance were *Haemaphysalis leporispalustris*, *Ixodes dentatus*, and *I. scapularis*, but 2 neotropical tick species of the genus *Amblyomma* were sampled during the spring migration. *I. scapularis* ticks were absent at the beginning of the study but constituted the majority of ticks by study end and were found predominantly on birds captured in areas designated as urban green spaces. Of 120 ticks, 5 were infected with *Borrelia burgdorferi*, spanning 3 ribotypes, but none were infected with *Anaplasma phagocytophilum.* Results allow inferences about propagule pressure for introduction of tick-borne diseases and emphasize the large sample sizes required to estimate this pressure.

## Abstract 2

No longer do you have to visit rural areas to find ticks; birds are flying them directly to you. When researchers sampled several thousand birds in Chicago, they found that some carried ticks and that some of these ticks carried the organism that spreads Lyme disease. Although the number of infected ticks on these birds was low, risk for their invading an area and spreading infection to humans cannot be ignored. If conditions are favorable, a few infected ticks can quickly multiply. Migratory birds also carried tick species only known to be established in Central and South America. Limited introduction and successful establishment of ticks and disease-carrying organisms pose a major health risk for humans, wildlife, and domestic animals in urban environments worldwide.

Wild birds can affect zoonotic disease risk to humans, wildlife, and domestic animals through their mobility and influence on the distribution and abundance of pathogens and vectors. Most notably, avian migration allows for rapid transcontinental transportation of novel pathogens and vectors that may seed new disease foci in receptive environments. For example, the spread of highly pathogenic avian influenza into and throughout most countries in Europe most likely occurred through the movement of migratory birds (*1*). Infected wild birds also contributed to the spread of West Nile virus (WNV) across North America (*2*). Thus, models of interseasonal connectivity among areas used by migratory birds can be used to forecast disease spread (*3*).

Over finer spatial scales, the patterns of bird use by blood-feeding vectors affect the prevalence of vector-borne pathogens. Host variation impacts the survival of vectors that feed on birds rather than on other vertebrates (*4*), and avian species exhibit differential reservoir competency for vector-borne pathogens (*5*). In combination, these factors influence disease risk; for example, just a few avian species that are heavily fed upon by mosquitoes and highly competent for WNV apparently drive most WNV transmission (*6*). Furthermore, host association of strains might help maintain pathogen diversity in some vector-borne diseases systems for which birds play critical roles (*7*).

Urban environments may promote pathogen transmission through increased host contact rates, high rates of pathogen introduction (i.e., propagule pressure), and warmer microclimates that are favorable to pathogens and vectors (*8*). These effects, in turn, may elevate disease risk to high-density urban human populations. Across gradients of urbanization, the incidence of some zoonotic pathogens has been found to be highest in urban cores (*9*). Reduced species richness in urban areas may contribute to elevated risk for diseases that are caused by multihost pathogens with generalist vectors (*10*), although the associations between biodiversity and disease risk are variable (*11*).

In humans, Lyme disease and anaplasmosis caused by infection with the bacteria *Borrelia burgdorferi* and *Anaplasma phagocytophilum*, respectively, are the 2 most common tick-borne diseases in the midwestern and northeastern United States, and both are emerging among human and canine populations (*12*,*13*). In eastern North America, both pathogens are maintained in blacklegged tick (*Ixodes scapularis*)–rodent cycles (*14*,*15*). We investigated the role of birds in the urban ecology of tick-borne zoonotic diseases. Our objectives were to 1) ascertain the prevalence of tick parasitism of birds in residential and urban green spaces in southwestern suburban Chicago, Illinois, USA, during a 6-year period; 2) estimate the infection prevalence of *Borrelia* spp. and *A. phagocytophilum* in ticks removed from birds; and 3) characterize the diversity of pathogens in ticks removed from birds by using genetic methods.

## Materials and Methods

### Bird Capture

During May–October 2005–2010, birds were captured at 20 field sites in southwestern suburban Chicago (Cook County; 87°44′ W, 41°42′N; Figure). Field sites were categorized as residential sites (n = 14) or urban green spaces (n = 6) and have been described in detail (*6*). We used 8–10 mist nets (Avinet, Dryden, NY, USA) to capture birds at 7–15 sites per year ≈1 morning per site every 1.5 weeks (2005–2007) or every 3 weeks (2008–2010). For each captured bird, we recorded species, sex, age class (hatch year and after hatch year), and weight, and we attached a numbered leg band before release. All birds were checked for ticks by blowing apart feathers and inspecting the skin, especially around the ears, head, and vent. Ticks were removed and preserved in 70% ethanol. Migratory status of each avian species was assigned (*16*). Fieldwork was carried out with approvals from animal care review boards at Michigan State University and University of Illinois.

**Figure:**
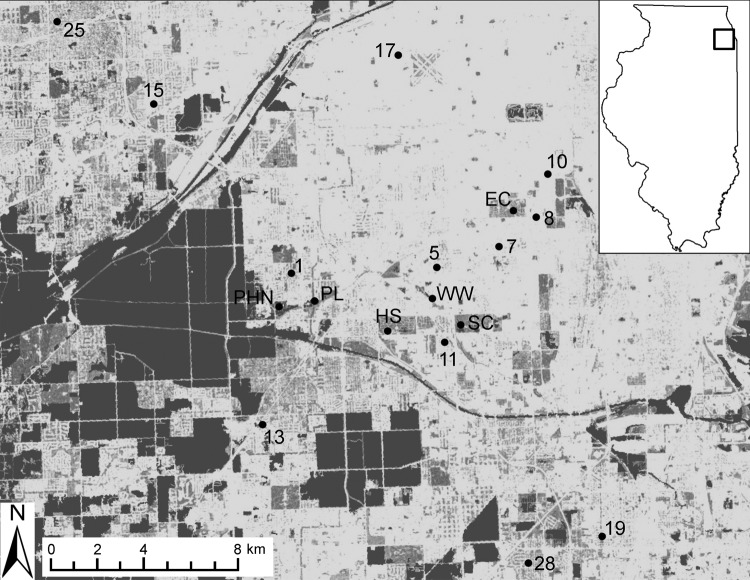
Field sites used for sampling birds in southwest suburban Chicago, Illinois, USA, 2005–2010. Sites consist of residential areas (numbered sites) and urban green spaces (lettered sites). Two residential sites not shown on the map (21 and 22) are ≈20 km north of this region. Box in inset map indicates location of sampling area. Main map shows the landscape gradient of impervious surfaces (National Land Cover Database 2001, US Geological Survey, Sioux Falls, SD, USA): dark gray areas are those with a low proportion of impervious cover (urban green spaces, e.g., forest preserves, parks, cemeteries, riparian buffers); light gray areas and white areas are those with a high proportion of impervious cover (areas with high density of buildings, residential housing, and roads). EC, Evergreen Cemetery; PHN, Palos Hills Natural; PL, Pleasure Lake; WW, Wolfe Wildlife Refuge; HS, Holy Sepulchre Cemetery; SC, Saint Casimir Cemetery.

### Detection and Typing of *Borrelia* spp. and *A. phagocytophilum*

Ticks were identified morphologically to species and stage; a subset was subjected to PCR and sequencing for confirmation (*17*). All ticks were tested for pathogens, except for 2 specimens that were deposited in the US National Tick Collection (housed at Georgia Southern University, Statesboro, GA, USA) for molecular identification and vouchering. Total DNA from ticks was extracted by using a DNeasy Blood and Tissue Kit (QIAGEN, Valencia, CA, USA) with modifications as described (*18*). Nymphal ticks were extracted individually, whereas same-species larvae from the same individual animal were pooled. All ticks were tested for the presence of *B. burgdorferi* sensu stricto and *A. phagocytophilum* by using a quantitative PCR targeting the 16S rRNA gene (*19*) and PCR targeting the *p44* gene (*20*), respectively.

*B. burgdorferi–*positive tick samples were typed by DNA sequencing of both strands of the 16S–23S rRNA intergenic spacer (IGS) region (*21*); strains were identified, using updated nomenclature (*22*), to ribosomal spacer type 1, 2, or 3 (*23*) and IGS subtype by comparing them with the 25 major *B. burgdorferi* IGS subtypes (*21*,*24*). The outer surface protein C (*ospC)* genotype was inferred on the basis of the linkage disequilibrium between IGS locus and *ospC* locus (*21*,*22*).

### Statistical Analyses

Logistic regression was used to assess the variation in tick infestations among years. We used 2- and 3-sample tests for equality of proportions to assess the effects of site category, sex, and age on the prevalence of tick infestations. The Wilson interval with continuity correction was used to estimate the 95% binomial CIs for infection prevalence data. Minimum infection prevalence (i.e., assuming 1 positive larva/pool) was used for tests conducted on pooled larvae. Statistical analyses were performed by using Program R (R Foundation for Statistical Computing, Vienna, Austria).

## Results

### Bird Captures

We recorded 6,180 total captures, comprising 5,506 individual birds (10.9% recaptures) and 78 species (Table 1). Five species comprised 67% of all captures: *Passer domesticus* (house sparrow), *Turdus migratorius* (American robin), *Dumetella carolinensis* (gray catbird), *Spinus tristis* (American goldfinch), and *Cardinalis cardinalis* (northern cardinal). Among all captured birds, 27.3% were known males, 21.3% known females, and 51.3% of unknown sex. The age class was after hatch year for 53.1%, hatch year for 41.8%, and unknown for 5.1% of the birds. Similar numbers of birds were captured from residential sites (3,326, 53.8%) and urban green spaces (2,854, 46.2%). Approximately 2× the number of birds were captured per year in 2005–2007 (1,455 ± 45) as in 2008–2010 (605 ± 159) due to higher mist netting efforts in the initial 3 years of the study.

**Table 1:** Birds sampled for presence of ticks in southwestern suburban Chicago, Illinois, USA, 2005–2010*

Bird	Migratory status	Total no. examined	Proportion infested	No. birds infested with						
				*Haemaphysalis leporispalustris*			*Ixodes dentatus*		*I. scapularis*	
				Larvae	Nymphs		Larvae		Larvae	Nymphs
American goldfinch	B, M	363								
American redstart†	B, M	38	0.03							
American robin	B, M	1,049	0.01	2	4		1		4	2
Baltimore oriole	B, M	31								
Barn swallow	B, M	7								
Black and white warbler	NB, M	9								
Black-capped chickadee	B, NM	25								
Blue jay	B, M	22	0.09							2
Brown-headed cowbird	B, M	65								
Brown thrasher	B, M	12								
Cedar waxwing	B, M	16								
Chipping sparrow	B, M	24								
Common grackle	B, M	105	0.03		2		1			
Common yellowthroat	B, M	8								
Dark-eyed junco	NB, M	8								
Downy woodpecker	B, M	50								
Eastern wood-pewee	B, M	5								
*Empidonax* spp. flycatchers	B, M	27								
European starling	B, M	141	0.01	1						
Fox sparrow	NB, M	5								
Gray catbird	B, M	429	0.01	3	3					
Gray-cheeked thrush	NB, M	18	0.11	1						1
Hermit thrush	B, M	5								
House finch	B, M	157								
House sparrow	B, NM	2,097	0.01	25	4					
House wren	B, M	57	0.02	1						
Indigo bunting	B, M	19								
Least flycatcher	B, M	5								
Lincoln's sparrow	NB, M	5								
Magnolia warbler	NB, M	19								
Mourning dove	B, M	63								
Mourning warbler	NB, M	5								
Nashville warbler	NB, M	7								
Northern cardinal	B, NM	311	0.04	9	3				1	
Northern flicker	B, M	10								
Northern waterthrush	NB, M	44								
Orchard oriole	B, M	4								
Ovenbird	B, M	41	0.10	4						
Palm warbler	NB, M	6								
Red-eyed vireo	B, M	11								
Red-winged blackbird	B, M	191	0.01	1	2					
Song sparrow	B, M	228	0.07	13	6		1			
Swainson's thrush‡	NB, M	131	0.08	4	4		1		1	
Tennessee warbler	NB, M	9								
Tree swallow	B, M	14								
Veery	B, M	8								
Warbling vireo	B, M	35								
White-crowned sparrow	NB, M	11								
White-throated sparrow	NB, M	61	0.02				1			
Willow flycatcher	B, M	63								
Wilson's warbler	NB, M	8								
Yellow warbler	B, M	34								
Yellow-bellied flycatcher	NB, M	6	0.17				1			
Yellow-rumped warbler	NB, M	26								
All		6,197§	0.02	64	28		6		6	5

*Empidonax spp. flycatchers that could not be identified are considered at the genus level. Numbers of birds infested by larvae and nymphs of 3 tick species are indicated. Common names conform to species as specified by the American Ornithologist Union. B, confirmed breeding in Chicago region; M, migratory; NB, non-breeder in Chicago region; NM, non-migratory. Blank spaces mean none infested. †One American redstart infested with a single *Amblyomma longirostre* nymph. ‡One Swainson's thrush infested with a single *A. nodosum* larva. **§**This total includes 49 unlisted captured birds from the following species: American woodcock, American tree sparrow, black-billed cuckoo, black-throated blue warbler, blackpoll warbler, brown creeper, Carolina wren, Canada warbler, Eastern towhee, Eurasian collared–dove, great crested flycatcher, golden-crowned kinglet, hairy woodpecker, killdeer, marsh wren, olive-sided flycatcher, red-breasted nuthatch, rose-breasted grosbeak, ruby-crowned kinglet, savannah sparrow, scarlet tanager, swamp sparrow, white-breasted nuthatch, and wood thrush. The sample size for each of these species was <5, and none of the birds harbored ticks.

### Tick Prevalence

We removed 357 ticks from 97 individual birds (1 bird with ticks was caught twice), yielding an overall tick infestation prevalence of 1.6% (Table 1). Ticks were usually located beneath the auricular feathers within the skin of the ear canal and second most commonly located in the rictus of the bill and in the skin of the orbital region. Infested birds were collected at 17 of the 20 field sites (11/14 residential sites, 6/6 urban green spaces). Birds with the highest prevalence of infestation (>7% of captures infested) were song sparrows (*Melospiza melodia*), Swainson’s thrushes (*Catharus ustulatus*), blue jays (*Cyanocitta cristata*), ovenbirds (*Seiurus aurocapilla*), gray-cheeked thrushes (*Catharus minimus*), and yellow-bellied flycatchers (*Empidonax flaviventris*) (Table 1).

Most ticks were of 3 species: *Haemaphysalis leporispalustris* (87.4% of all ticks), *Ixodes dentatus* (4.8%), and *I. scapularis* (7.8%). Morphologic and molecular identifications were congruent for all 21 birds subjected to both methods of identification (GenBank accession nos. JQ868565–JQ868585). Overall, 1.3%, 0.1%, and 0.2% of birds were infested with *H. leporispalustris*, *I. dentatus*, and *I. scapularis*, respectively (Table 1). In addition, a single *Amblyomma nodosum* larva was removed from an after–hatch year Swainson’s thrush on May 17, 2005, and a single *A. longirostre* nymph was removed from an after–hatch year American redstart (*Setophaga ruticilla*) on May 18, 2005. The 2 ticks were found on birds captured at site HS (see Figure) during the spring migration. They were identified genetically and vouchered at the US National Tick Collection but not tested for pathogens.

The number of ticks on infested birds ranged from 1 to 23 (median 2 ticks). Of the infested birds, 47% harbored 1 tick and 20% harbored >5 ticks. *H. leporispalustris* larvae accounted for the greatest tick loads (average 4.3 ticks/bird). Of 98 parasitized birds, 11 (11.2%) were infested with >1 life stage of tick or >1 tick species. Although the overall prevalence of infested birds did not change over the 6-year study (z value = −1.6, *df* = 6178, p = 0.109), the proportion of infested birds that harbored *I. scapularis* increased significantly from 0 to 80% (z value = 3.873, *df* = 96, p = 0.0001), and *I. scapularis* comprised >90% of ticks removed from birds in the final year of the study. Of the 10 *I. scapularis*–infested birds, the majority (8) came from urban green spaces (0.28% *I. scapularis* infestation prevalence across all green spaces), and the minority (2) came from residential sites (0.06% prevalence; z value = 2.2, p = 0.03). Information about the timing of *I. scapularis* infestation combined with the species and age of the avian host provides evidence for local (Chicago area) acquisition of ticks and for migratory importation of ticks from the north and the south (Table 2).

**Table 2:** Demographic information about 10 avian hosts infested with *Ixodes scapularis* ticks in southwestern suburban Chicago, Illinois, USA, 2005–2010*

Bird	Date of capture	Age	Site, category	*I. scapularis* stage (quantity)	Presumed *I. scapularis* acquisition
American robin	2007 Jul 18	AHY	1, residential	L (9); N (1)	Local
American robin	2009 Aug 18	HY	PL, green space	L (2)	Local
American robin	2010 Jun 22	AHY	PHN, green space	N (2)	Local
American robin	2010 Jul 13	AHY	PL, green space	L (1)	Local
American robin	2010 Jul 26	HY	PL, green space	L (8)	Local
Blue jay	2009 Jun 15	AHY	PHN, green space	N (1)	Local
Blue jay	2009 Jun 15	AHY	PHN, green space	N (1)	Local
Gray-cheeked thrush	2010 Sep 16	HY	PHN, green space	N (1)	Migratory (from north)
Northern cardinal	2007 Aug 16	HY	13, residential	L (1)	Local
Swainson’s thrush	2006 May 23	AHY	WW, green space	L (1)	Migratory (from south)

*AHY, after hatch year; L, larva; N, nymph. HY, hatch year; PL, Pleasure Lake; PHN, Palos Hills Natural; WW, Wolfe Wildlife Refuge.

### Tick Infection with *B. burgdorferi* and *A. phagocytophilum*

A total of 120 tick samples were tested for pathogens. No ticks tested positive for *A. phagocytophilum* infection. Five samples tested positive for *B. burgdorferi* infection: 3 of 6 *I. scapularis* nymphs (50%, 95% CI 14.0%–86.1%), 1 of 22 *I. scapularis* larval pools (minimum infection prevalence 4.5%), and 1 of 34 *H. leporispalustris* nymphs (2.9%, 95% CI 0.2%–17.1%) (Table 3). All 5 positive tick samples were from unique after–hatch year birds of 4 species (American robin, blue jay, red-winged blackbird [*Agelaius phoeniceus*], Swainson’s thrush) at 4 field sites, including urban green spaces and residential sites. *B. burgdorferi* 16S–23S rRNA IGS sequences were obtained from all 3 *I. scapularis* nymphs and represented 3 IGS ribotypes (2, 28, and 14; GenBank accession nos. JQ868562–JQ868564) within ribosomal spacer type 2 and 3; inferred *ospC* genotypes were H, T, and A3, respectively (Table 3).

**Table 3:** Prevalence of *Borrelia burgdorferi* infection in ticks removed from birds, by site of origin and date of capture, southwest suburban Chicago, Illinois, USA, 2005–2010*

Tick species	Larva				Nymph				
	No. pools (no. larvae)	% Infected (MIP)	Birds with infected larvae, site, date		No. tested	% Infected (95% CI)	Birds with infected nymphs, site, date	IGS strain (RST group)	*ospC* strain
*Haemaphysalis leporispalustris*	65 (277)	0	NA		34	2.9 (0.2–17.1)	RWBL, SC site, 2007 Jun 6	NA	NA
*Ixodes dentatus*	6 (17)	0	NA		0	NA	NA	NA	NA
*I. scapularis*	6 (22)	16.7 (4.5)	SWTH, WW site, 2006 May 23		6	50 (14.0–86.1)	AMRO, 1 site, 2007 Jul 18; AMRO, PHN site, 2010 Jun 22; BLJA, PHN site, 2009 Jun 15	2 (2); 28 (3); 14 (2)	H, T, A3

*MIP, minimum infection prevalence; IGS, *B. burgdorferi* 16S-23S rRNA intergenic spacer ribotype; RST, ribosomal spacer type 1, 2, or 3; *ospC*, inferred outer surface protein C allele based on linkages reported by Travinsky et al. (*23*); NA, not applicable; RWBL, Red-winged blackbird; SC, Saint Casimir Cemetery; SWTH, Swainson’s thrush; WW, Wolfe Wildlife Refuge; AMRO, American robin; PHN, Palos Hills Natural; BLJA, Blue jay.

## Discussion

The presence of *B. burgdorferi*–infected *I. scapularis* ticks on migratory and residential birds in the Chicago region reflects the continued invasion and establishment of this tick and pathogen across the Midwest. In Illinois, as in many other areas of North America (*25*), there is growing public health concern over the emergence of Lyme disease (*26*); although, the statewide incidence in Illinois over the study period (1.1 cases/100,000 persons) was an order of magnitude lower than that which characterizes the Lyme disease–endemic regions in the northeastern United States (*27*). Our study provides evidence of established local populations of *I. scapularis* ticks in Chicago that may be supplemented by importation of *I. scapularis* ticks from other populations to the north or south by migratory birds. The Chicago region is a natural corridor for migratory birds, and the risk for tick and pathogen introduction is likely to be elevated on migratory flyways because of seasonal concentrations of birds.

We detected a *B. burgdorferi*–positive *I. scapularis* larval pool from a Swainson’s thrush. Given the absence of transovarial transmission in the *I. scapularis* tick, this finding demonstrates that the Swainson’s thrush can be an infectious reservoir host. On the basis of a limited sample (n = 6), we determined that birds in Chicago harbored *B. burgdorferi*–infected *I. scapularis* nymphs at a prevalence (14.0%–86.1%) consistent with that reported for questing nymphs and ticks from birds in Michigan (*18*), Minnesota (*28*), and Canada (*29*). All 3 *B. burgdorferi* IGS ribotypes present within nymphs in this study have been associated with host-seeking nymphs in Lyme disease–endemic areas of the midwestern and northeastern United States; 2 of the 3 ribotypes were previously detected in larvae removed from birds (*30*). Two of the *ospC* types (H and A) presumed present in the collected ticks were among the 4 most invasive genotypes (I, A, H, B) from a study of *B. burgdorferi* isolates from humans in New York (*31*). The presence of avian reservoirs and *I. scapularis* nymphs infected with *B. burgdorferi* strains capable of causing disseminated human disease supports the possibility that reported cases of human Lyme disease in Chicago residents may result from local exposure to infected *I. scapularis* ticks. Although none of the ticks removed from birds were positive for *A. phagocytophilum*, the growing *I. scapularis* tick population in the region raises the possibility that infection with this pathogen could become an emerging health concern.

Other ticks commonly found on birds in Chicago are *I. dentatus* and *H. leporispalustris* ticks, both of which feed almost exclusively on rabbits and birds. *I. dentatus* ticks are enzootic vectors of *B. burgdorferi* in regions where *I. scapularis* ticks do not occur (*24*). *H. leporispalustris* ticks transmit *Francisella tularensis* and spotted-fever group rickettsiae among wildlife (*32*). In our study, *H. leporispalustris* ticks had a wide geographic presence across most residential sites and were most commonly found on house sparrows, including 7 hatch-year birds, implying local acquisition in the residential neighborhoods. Neither *I. dentatus* nor *H. leporispalustris* ticks regularly infest humans.

We document the presence of 2 neotropical tick species, *A. longirostre* and *A. nodosum*, on birds migrating north through Chicago. We note that other species of neotropical *Amblyomma* ticks have been recovered in the spring on migrant birds in southern Canada (*33*). *A. longirostre* and *A. nodosum* ticks are widely distributed in the neotropical region, and are vectors of *Rickettsia amblyommii* (*34*) (which may cause rickettsiosis in humans in North America) (*35*), *R. bellii*, and *R. parkeri* (*36*). In the United States, *R. parkeri* is a newly recognized cause of human disease, and a high prevalence of infection (>40% in adults) has been associated with growing populations of Gulf Coast ticks (*A. maculatum*) (*37*). Migrant birds from the neotropics likely account for many imports of engorged neotropical ticks and associated pathogens in North America each spring, but a lack of environmental receptivity (host or climatic limitations) has likely prevented establishment.

Data from our large sampling effort show that the dispersal of *I. scapularis* ticks, *B. burgdorferi*, and neotropical vector ticks is a rare but detectable event. We sampled several thousand birds and detected *I. scapularis* ticks on <0.2% and neotropical ticks on <0.05%. However, the rarity of infestations does not mean that infestation is biologically insignificant. Despite the positive relationship between propagule pressure and invasion success, some successful species invasions, especially those of arthropods, can be initiated by a very small number of individuals (*38*). Low propagule pressure but successful invasion may occur when the environment is receptive to the particular species of ticks and pathogens being dispersed. Indeed, during our study, other researchers showed an increase in the occurrence of *B. burgdorferi*–infected adult *I. scapularis* ticks in northwestern Chicago, confirming our prediction (*26*). Such scenarios of rare introduction but successful establishment of ticks and pathogens pose a major risk for the health of humans, wildlife, and domestic animals in urban environments worldwide.
